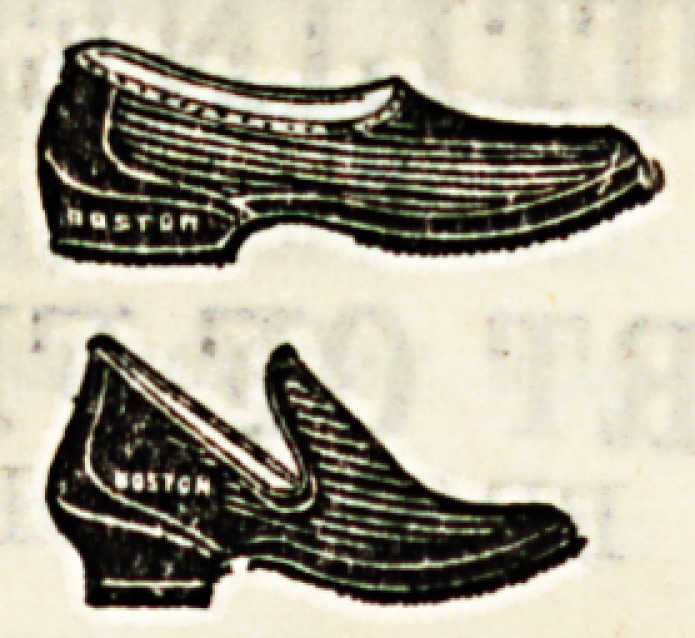# The Hospital Nursing Supplement

**Published:** 1894-12-29

**Authors:** 


"The Hospital" Nursing Mirror.

Being the Extra Nursing Supplement of "The Hospital" Newspaper.

[Contributions for this Supplement should be addressed to the Editor, The Hospital, 428, Strand, London, W.C., and should have the word "Nursing" plainly written in left-hand top corner of the envelope.]

### News from the Nursing World.

### OUR PRINCESS.

It cannot fail to interest the many readers of The Hospital who last week welcomed the portrait of H.R.H. the Princess of Wales, to learn it was so highly appreciated at Marlborough House that H.R.H. the Prince of Wales wrote for a second copy of our last issue, saying he had forwarded the first by special messenger to the Princess at St. Petersburg, as he desired her to have it on Christmas Day, and wished to keep one for himself. It is not therefore to be wondered at that many letters of congratulation and thanks have reached us from all parts of the country, testifying to the esteem and regard in which the Princess of Wales is universally held by all classes. Her work both in actual nursing and in the interest of nurses, was forcibly brought forward last week in the sketch of "Our Princess" which accompanied her portrait.

### A HAPPY NEW YEAR.

We have to thank many nurses—more than we can personally reply to—for kind wishes and seasonable greetings. We like to read appreciative acknowledgments of the services rendered by The Hospital during the years in which it has steadily obtained increased popularity. "I don't know what I should do without my Hospital," said the head of a large nursing staff. "I always find any nursing news worth hearing in The Hospital" writes another. Our readers must not fancy that we are indifferent to encouragement, or that we feel too busy to be pleased by kind letters. Again we urge upon nurses, far and near, that news sent in by themselves is peculiarly valuable. News or information is not necessarily gossip, the latter being as worthless as the former is worthy. Reliable notes from trustworthy sources are always appreciated by our fellow-workers, and the promotion of progress is best secured by constant interchange of reported improvements in every department of skilled nursing. As the old year has been successful, so, dear readers, please aid us to make the new one still better, and in helping each other we shall, perhaps unconsciously, each help ourselves to enjoy A Happy and Bright New Year!

### GUY'S HOSPITAL NURSES.

Last week we noticed the recent announcement made at Guy's Hospital respecting the pensions of the nursing staff. In the days when there was no Royal National Pension Fund to join, and even after its establishment, when many failed to realise the great future which lay before that wonderful scheme, due provision was made by this hospital for its aged and disabled workers. But at the present time it is obvious that the Fund renders any other system of pensions unnecessary, and the authorities of Guy's Hospital have therefore decided to discontinue their former plan. The sisters, nurses and probationers are encouraged in every way to provide for their own future, their policies being most liberally supplemented by the hospital. Assistance in thriftily helping themselves is the only aid which is palatable to independent and self-respecting nurses.

### "A NECESSITY AND A LUXURY."

Miss Spring's work at Hampstead during the eleven years in which she has been Superintendent of the Nursing Association, was pleasantly acknowledged the other day at the social evening arranged on the occasion of her giving up her post. The testimonial presented to her was subscribed for entirely by former patients who had been under her care in Gospel Oak, Kentish Town, Hampstead, and Kilburn, a number of these poor people being guests at the tea party which formed the first item in the evening's proceedings. The Vicar of St. Stephen's presided, and in a kindly speech remarked that trained nurses were the most valuable discovery of the present day. They were both "a necessity and a luxury." A handsomely fitted English-made desk was the gift purchased by the poor for their friend, and great regrets were expressed on all sides at the prospect of their losing her. From a letter signed G. Hay Reynolds, M.B., in the local press, it appears that Miss Spring's resignation is solely due to the inadequate financial local support given to the Nursing Association.

### SANTA CLAUS SOCIETY.

The Santa Claus Society has held its tenth annual exhibition at Highgate, under the management of the Misses Charles. A capital collection of toys, dolls, and useful clothing was shown to a numerous party of visitors; and the things were distributed as usual to St. Pancras Infirmary, All Saints' Convalescent Home, Holborn Infirmary, Cromwell House, the Great Northern and the North-West London Hospitals, the North London Nursing Association, and the Santa Claus Home at Highgate.

### WHO MAKES THE BEDS?

The practice of making patients' beds in the small hours of the morning has become obsolete in most institutions, but unfortunately not quite in all. To condemn nurses who have been on duty all night to conclude their duties with such work is cruel to them and to their sick charges. The custom is a relic of a period when the "sitter-up" was employed. She was a woman unskilled in nursing who was set to make beds in the morning after a quiet night spent in an easy chair! Now-a-days the temptation to get ahead with an onerous duty is obvious, and the conscientious nurse finds justification for beginning a "heavy case" at four a.m. because later on it would be impossible for her to devote sufficient time to "doing his bed comfortably." Thoughtless women do not restrict themselves to heavy cases, rousing patients at hours little dreamt of by the medical staff. Of course surgical and accident patients, often, as they acknowledge, "quite well in themselves," awake at hours to which custom has familiarised them, and the majority of ward patients are asleep before nine p.m., but no one should be victimised, as is the case when bed-making ranks amongst the prerogatives of the night nurse.

### FALSE OR TRUE?

If the criticisms on the hours and work of the nursing staff at Newcastle Infirmary have no justification of truth, it would be well for a prompt authoritative assurance on the subject to be publicly given. If, on the other hand, a need for alteration exists, surely a frank acknowledgment and an adequate reform would meet the case better than any newspaper controversy. Sympathy is unfortunately often tinged by sickly sentiment, and the "disagreeable duties" performed by "young girls" provoke remarks which lack common sense. We are inclined to pity patients who have such youthful attendants rather than the "girls," whose parents should forbid their entering on a nursing career until they have arrived at suitable ages for serious responsibilities. The public discussion on the washing of patients shows how little is understood on such practical points by those outside hospitals; while the ablutions of helpless persons are necessarily performed by nurses, the bathing of men, to which so much attention has been called, is invariably done by porters or other suitable persons, in all properly conducted institutions.

### UNFED AND UNNURSED.

Whether in or out of hospital, food is a matter which cannot be lightly considered at any time, and just at this season it is very much in evidence. Whilst tempting the uncertain appetite of chronic or fanciful invalids, the art of sick-room cookery and the preparation of all forms of condensed nourishment come constantly before private and hospital nurses. The infirmary nurse also finds that the Guardians seldom grudge suitable diets to "urgent" cases, but between the kitchen and the patient in the sick wards of some workhouses there exists the formidable barrier of "pauper helps." At Penzance this has resulted in an old man, apparently dying, refusing his food for three days. As "no one appeared to be told off to attend to him," according to the local press, it is easy to understand the so-called refusal in the face of no trained night or day nurse being employed at this workhouse, where the sick, aged, and dying are in the care of fellow paupers, supervised by one untrained paid female officer.

### WHERE WAS THE THERMOMETER?

A young woman, aged 22, engaged as a probationer at Wirral Infectious Diseases Hospital, has been accused of causing the death of a poor little child of two or three years old. The occurrence is lamentably sad and cannot be characterised as merely "accidental," for if the report in the local press is accurate, the mode in which the fatal bath was given was incredibly careless. The heat of the water was not tested in any way, according to the nurse's own confession, before the little patient was placed in it, the result being such terrible scalds that death resulted the following day. It is to be hoped that the adjourned inquiry will show that the thermometer is not usually omitted at Wirral in the preparation of the baths, which are important features in the treatment of infectious diseases.

### ST. ELIZABETH'S HOME.

At Glasgow a meeting of the Ladies' Assistant Committee of St. Elizabeth's Home was held early in December. Bishop Maguire presided, and spoke of the work done by these lay sisters of charity amongst the sick poor. Private nursing is also undertaken by the Home, which was established by Lady Bute, and the co-operation of "the Catholic ladies of Glasgow" is cordially invited. Owing to the colour of their dress, the sisters are usually designated "the Grey Nurses."

### PRIVATE PATIENTS IN AFRICA.

Miss Rose Blennerhassett's work in England and Africa is well known to our readers, and what she and Miss Sleeman have accomplished in the island of St. Helena is also becoming equally familiar to sister-nurses at home. Her letter in another column of this paper shows the new scheme to which she proposes next month to devote herself, and should it succeed, as it ought, this new departure will be as noteworthy as any past deeds of the two energetic friends. A nursing home in South Africa appears to be urgently demanded by the numerous invalids sent out from England in search of health, a quest grievously hindered by the hardships in the colonies. Should Miss Blennerhassett and Miss Sleeman prove the feasibility of establishing a comfortable home, with poultry and cows attached in the establishment, green vegetables and fruit equally attainable, the two enterprising nurses will deserve gratitude from many invalids.

### A NOBLE NEW YEAR'S GIFT.

"I can never repay her kindness," sighed the convalescent patient, "but I want her to have some substantial proof of my gratitude. What can I do?" The friend considered the matter thoughtfully. "Could you take out a policy for her in the Nurses' National Pension Fund? I know your nurse was saying the other day that she should always regret not having joined it at first, but she did not understand what a grand scheme it was; since then circumstances have combined to hinder her paying in. Take out a policy by all means, and make a New Year's gift of it." "And set employers of nurses a good example into the bargain? Thanks for the hint. When one has come back from the very gates of death one's 'thank-offering' may well include gifts to the doctor and nurse."

### SHORT ITEMS.

It is proposed to affiliate the new nursing association at Barrhead with the Queen Victoria's Jubilee Institute.—Miss Johnstone has been made Superintendent of the District Sick Nursing Home, Edinburgh.—A successful bazaar at Devizes has taken place in aid of the funds of the Mothers' Nursing Home.—A concert given at Dursley High School for Boys resulted in a donation of £5 to the Dursley Nursing Association.—After payment of all expenses, £100 was handed to the Helena Nursing Home at Reading, being proceeds of the sale of work held on its behalf in the town.—An excellent article on "Trained Nursing in America," by Miss Darche, Superintendent of New York City Training School, appears in the December number of *The Trained Nurse.* It is illustrated with numerous pictures of nurses in the uniforms of various hospitals.—It is proposed to raise a fund to support a district nurse as Elsham, as a memorial to the late Sir John Astley.

### The Dietetic Management of Infancy.

By GEORGE DICKSON, M.B., C.M.

### III.—FEEDING BOTTLES AND FOOD.

### THE BOTTLE.

A HEALTHY mother gives her child during the earlier months of lactation about one pint in twenty-four hours, increasing later to about three pints. This quantity should therefore act as a standard, and either of the following meals will generally do well for any child under one month old: (1) Barley water, three tablespoonfuls; milk (boiled), one tablespoonful. (2) Water, three tablespoonfuls; gelatine, one teaspoonful; milk (boiled), one tablespoonful. (3) Water, one tablespoonful; lime-water, two tablespoonfuls; milk (boiled), one tablespoonful. To each of the above about half a teaspoonful of sugar should be added. Milk sugar probably possesses little advantage over the ordinary kinds.

It is very evident that in nutritive value a pint of any of the above mixtures is very considerably inferior to a pint of healthy human milk, but in actual practice children are found to thrive quite well, at least for a time, on about that quantity of any of the formulæ given, and if well tolerated, the strength can be readily increased.

There are two kinds of feeding-bottles, and it is by no means a matter of indifference which is selected. In one the teat is attached to a long india-rubber tube, fastened to a glass tube which reaches to the bottom of the bottle. In the other the teat is put on the neck of the bottle without any tubes. Bottles with tubes are popular because the child using them does not require to be taken up when fed, but the practice of leaving an infant to take his nourishment by himself is an objectionable one, often productive of evil consequences.

The child "bolts" the food, the bottle is quickly emptied, and air sucked in, which gives rise to flatulence. When it is remembered that a child's stomach shortly after birth is only capable of containing three tablespoonfuls of fluid it will be understood that the organ is easily overloaded.

A great disadvantage of tubes is the impossibility of keeping them clean. The small brushes usually sold for the purpose are dangerous on account of the facility with which the bristles became detached and liable to stick in the child's throat.

The advantage of the tubeless bottles lies in the simplicity of their construction, and the ease with which they can be cleansed. Moreover, it is absolutely necessary that the child should be taken up whilst having the bottle. The soda water shape is a good one, and there should always be two, used alternately.

After thorough washing with hot water and soda they should be left to soak in cold water, to which has been added a pinch of borax or boracic acid.

It is impossible to over-estimate the importance of absolute cleanliness. The feeding may be perfect in every other particular, and yet, if the bottles are not thoroughly washed; if new tubes are not supplied when the old ones become unwholesome, as they always eventually do, flatulence, vomiting, or diarrhœa supervenes, and the whole process is rendered unsatisfactory. In the diagnosis of children's diseases it is well to examine the feeding-bottles, for in it is frequently to be found the seat of the mischief.

### THE FOOD.

An infant should be fed every two hours during the day for the first six weeks, and, as with breast-fed babies, not during the night, if this prove possible.

After six weeks the intervals should be extended to three hours, and the milk concentrated to one in three, the quantity given at a time being increased to three ounces. After this the quantity and strength should be gradually increased, careful watch being kept on the digestion, for the condition of the latter, more than the months of age, ought to be the chief guide. At the seventh or eighth month, if all has gone well, the child should be taking about six ounces (milk forming two-thirds of the mixture) to each meal every four hours. A tablespoonful of cream may be added to the bottle twice or thrice daily. Diluted milk should form the sole dietary up to this age, and two meals a day of wheaten flour or Mellin's food should afterwards be added, a cup being substituted for the bottle.

It is often necessary to wean a child earlier than twelve months. (1) If the milk supply is defective; (2) if the mother's health suffer from lactation; (3) if the mother become pregnant during lactation, and (4) if the mother become affected by intercurrent disease—General: Phthisis, pneumonia, typhoid, &c. Local: Abscess or other affection of the breasts.

In such cases the child should be fed as described under artificial feeding. It is never safe in beginning cow's milk for the first time, where the child is under eight months' old, to give a stronger concentration than one in three to start with. If it agree it can readily be increased as desired.

Where possible, the breast should continue to be given night and morning, or even once daily.

In cases of abscess of the breast it is sometimes possible to allow the child to suck at intervals from the sound breast until the affected gland recovers and the use of both can be resumed.

### INFANTILE DRAWBACKS.

Sucking may be rendered difficult or impossible from various more or less removable causes, such as tongue tie, cleft palate, thrush and other painful conditions of child's mouth, or congenital syphilis.

Tongue tie, in a degree sufficient to form an obstacle, is extremely rare, but cleft palate is more common, and with it sucking is physically impossible, and the infant requires to be fed with a spoon.

Thrush, &c., may cause a child to refuse the breast on account of the pain which sucking causes. Infants affected with congenital syphilis nearly always suffer from swelling of the nasal mucous membrane (snuffles), which prevents breathing through the nose when the month is closed.

When such infants take the breast they are forced to desist in order to breathe. In cases where the nipple is insufficiently drawn out a child may refuse the breast, not so much because it cannot grasp the nipple as because in doing so its face is hurt against the hardened body of the gland.

Wiping out the mouth frequently with glycerine of borax (one in four of water) will generally effect a cure in cases of thrush. The other conditions are best left to the doctor in charge.

### CONSTIPATION.

This is especially apt to be troublesome in bottle-fed babies. If breast-fed, the mother should take an occasional dose of salts or a seidlitz powder, and should increase the amount of fruit and vegetables in her meals. A hot bath in the morning, with or without subsequent massage of the abdomen, will often encourage an evacuation.

If bottle-fed, a dessertspoonful of fluid magnesia may be added to the morning meal. If the child be already taking starchy food, a teaspoonful of fine oatmeal rubbed up with the morning milk will generally act as a laxative. As an occasional aperient castor oil is often recommended.

### FLATULENCE AND COLIC.

These are common troubles in infancy. A child with colic cries loudly and passionately, drawing up his knees and kicking his legs about. If the pain be severe he may become quite collapsed. During sleep the muscles of his face twitch, often into the semblance of a smile. During the attack the feet should be rubbed with the warm hand alone or with warmed olive oil. A hot cloth, fomentation or poultice, should be applied to the abdomen, and the doctor may order ten drops of brandy in a dessertspoonful of dill, cinnamon, or caraway water, to be given internally. If there is constipation a dose of castor oil may be suggested. If flatulence becomes habitual, as it is apt to do in bottle-fed babies, it is well to make sure that the general conditions of artificial feeding are observed; and especially that the bottles are kept thoroughly clean. Barley water, lime water, or gelatin may be used as the diluting agent instead of plain water, and a tablespoonful of dill or caraway water may be added each bottle of food.

### Our Christmas Competitions.

All our Christmas parcels have been unpacked, and the contents admired, judged, sorted, and distributed. Each year we look forward to this as being one of our pleasantest duties. The warm garments are so pretty, and nurses, perhaps better than anyone else, can estimate the value of such woollen treasures to delicate men and women who necessarily leave behind them many bodily comforts in exchanging ward life for their ordinary home surroundings.

"No. 9 is discharged, and I asked his wife to bring his clothes and—look at them!" exclaims the nurse, to whose care No. 9's recovery is, to no small extent, due. If a good flannel shirt be forthcoming, and a warm pair of socks can be added to the worthy bread-winner's scanty outfit, nurse, patient, and wife rejoice to an extent undreamt of by people who have never been short of anything. The things sent in for our Christmas distribution, are, many of them, labelled "Not for competition"; and they are also noteworthy from being made by the hands of very busy women, and also as specimens of extremely good workmanship. The last fact is proved by the difficulty there has been found in allotting the prizes and this is, perhaps, more particularly true of the knitting. Really, many of the socks are beautifully made, both as regards shape and evenness of work. They are, moreover, valuable and acceptable gifts to hospital patients, and they find a hearty welcome on all sides. A great many fine stitches have been set in flannel and other petticoats, and the night-gowns are simply charming, especially those which Madame Monchablon and her friends (always most liberal contributors) sent in "not for competition." Their bed-jackets are also excellent in design and material, the breast pocket being an adjunct well appreciated by sick men, who find a bed, however small, quite big enough to lose things in. Most restless invalids realise the comfort of a handy place for the indispensable handkerchief, for which provision is often omitted in ordinary bed costumes. Madam Monchablon's boxes also contain scarlet flannel petticoats, black stockings, scarves, socks, knitted cuffs and gloves, a pretty frock in that excellent material Turkey twill, and some little petticoats. Nurse Wilson sent petticoats with bodices, and Nurse Edith two nice bonnets and a petticoat. From Miss A. H. Purvis have come boys' socks and a dainty jacket, and from Nurse Marjorie delicate white wool vests. Miss Agnes Gould sends children's bodices and petticoats; Miss Lunn, night socks; Miss Lockyer, knitted stockings and a woollen cross over; Mrs. Puddicombe, mufflers and a comforter; Miss Helen Grafton, socks, books, and respirators; "Two Hospital Readers" have sent beautiful flannelette night-gowns, which form very valuable gifts; and Nurse Bishop sent two warm hand-made Jerseys; from Sunderland came pretty baby vests; and from Sister Goalen, bodices, petticoats, and a knitted jacket.

Socks were received for the competition from Nurse Warren, Nurse M. Griffiths, Nurse Mackie, Nurse Lizzie, Nurse Pearce, Nurse Lowe, Miss Chisnall (two pairs), Nurse Emily Elliot (two pairs), Miss Isabel Allen, Nurse A. Bates, Nurse S. D. Curwood.

The first prize was won Nurse M. Griffiths for a very fine specimen of knitting. Nurse Emily Elliot (who kindly sen two pairs to ensure a change of socks to the recipient) and Nurse Mackie are equally skilled, therefore two second prizes had to be awarded.

The shirts marked for "competition" were really not quite as well made as those sent for distribution only, the best of the former being Miss Elphick's, to whom the prize was therefore awarded. Miss Delap and Nurse Bishop kindly sent useful shirts.

The first prize for a flannel petticoat was won by Nurse Prior, a second being adjudged to Mary H. Delap. Very nice petticoats were also sent in by Nurse L. H. Ulph, Nurse Reeve, and Policy 1,281. The prizes are always awarded by at least three judges, who bestow much care over their decisions. This year the selection was in the able hands of the matrons of St. Thomas's, Miss Gordon; Miss Nott-Bower, of Guy's Hospital; and Miss Moir, of The Infirmary, Dartmouth Park Hill.

No dressing-gowns, over-petticoats, or bed jackets were sent in for competition, but we acknowledge very gratefully the useful contributions sent in "for distribution."

Of all these things the supply can never equal the demand, for our sick poor are numbered by thousands in the London hospitals alone. All parcels sent are warmly welcomed by the recipients, not only for their practical value, but also as the achievements of busy workers who spare from their daily duties time to make "Christmas-boxes" for those who would otherwise get none.

Garments made by nurses always have a special interest, and they are in the present instance visible proofs of unselfish devotion and kindly sympathy with the sick poor who have spent Christmas Day, 1894, warded in hospitals.

To our helpers, those who with words of encouragement and appreciation, as well as to those who co-operate in our annual needlework competition, we wish a very glad and prosperous New Year.

Parcels have gone to the following hospitals: Westminster, University, Guy's, St. Thomas's, Middlesex, Charing Cross, the London, King's College Hospital, and the Royal Free, Gray's Inn Road.

From each institution a cordial acknowledgment has been sent, and we are asked to convey the thanks of the patients to those donors whose time and skill have been devoted to their service.

### Entertainments.

The subscription dance given recently in the Galleries of the Royal Institute of Painters, Piccadilly, will probably form the first of a series arranged by the students of University College and Hospital.

The first occasion on which the Picture Galleries at the Royal Institute have been used for dancing was "the Cinderella" recently given in aid of the funds of the Chelsea Hospital for Women.

An amateur dramatic performance was recently given at Digby Assembly Rooms in aid of the Sherborne Town Nurse Fund.

An entertainment at West Ham Hospital, arranged for 28th inst., to take place in the dispensary hall at eight p.m., offers an attractive programme to the guests.

At the National Hospital for Paralysis and Epilepsy a concert was given to the patients on the 13th inst., and was thoroughly appreciated by all who were able to be present.

Christmas Day at the Dreadnought Seamen's Hospital, Greenwich, began with morning service in the chapel at half-past ten for the convalescents, followed by dinner at half-past twelve. On December 28th the nurses' entertainment is arranged for half-past seven, Mr. Nairns (the deputy chairman) bringing his band, and on January 3rd the children's Christmas tree will be on view at half-past four.

Christmas Day is celebrated very pleasantly in Westminster Hospital, the patients being entertained in their respective wards. The decorations in the chapel looked particularly lovely, red and white flowers being chiefly used. Presents for the patients are distributed, and holly and other evergreens make the wards bright and gay.

The Annual Ball in aid of the funds of the Nottingham General Hospital took place last week, and was well attended and successful.

### Our American Letter.

(COMMUNICATED.)

WHEN this reaches you Christmas Day will be nearly over, perhaps, and HOSPITAL readers may imagine that their sisters in America have spent the festival in much the same way as themselves. Not at all! Over here we make much less of it than you do at home. Some people say we regard the day more exclusively from its religious aspect than you in England, and certainly we make it less of a feast than "Thanksgiving," which to you is an unknown date in the calendar. Anyway, we copy you (or, maybe, you imitate us?) in the matter of present-giving. In our hospitals we have introduced the time-honoured German Christmas tree for the entertainment of the smaller patients.

Miss Kimber's book will have reached you ere this. We think much of it here, and more still of the author. As a teacher she has done noble work, and as a woman and nurse her value can hardly be overpraised by those who have personal knowledge of her. She has held the position of assistant superintendent at New York City Training School for seven years, and has also had large experience in other hospitals.

An afternoon and evening reception was given by some ladies on the occasion of the opening of the new St. Barnabas Hospital, St. Paul, Minn. Numbers of visitors were present, and the buildings were inspected and admired.

Hitherto the offices of housekeeper and superintendent of training have been distinct at the Children's Hospital, Buffalo, N.Y., but recently both posts have been conferred on one lady, Miss Olivia Moore, who gives every promise of ably carrying out the double duties. It is quite a small establishment compared to English institutions, as there is accommodation for thirty-six children only. Seventy-nine were treated in the year preceding the issue of the last annual report, and when the little patients are discharged arrangements are made for continuing the treatment of the physicians.

A special free bed for sick newsboys or for those who sustain accidents has been instituted at the Children's Homœopathic Hospital, Philadelphia, Pa., and boys are invited to communicate with the president of the hospital, who will have their names registered in case illness or casualty renders their admission desirable.

A two-year course of training will be given to the pupils at the Nathan Littauer Hospital; and in connection with that hospital, situated at Gloversville, N.Y., a "Women's Auxiliary" has been formed. This association undertakes the hospital needlework, and pledges itself to do anything required to promote the welfare of the hospital or for the patients' benefit.

We wonder if similar associations are in force in any British towns, or if this practical form of assistance is peculiar to us at any rate it is.

There is accommodation for twenty-four nurses in the home connected with the Nurses' Association at Baltimore. This house is exclusively for graduates belonging to the association, and seems to be valued by them. A directory, organised and managed by the association, provides the public with trained nurses.

Perhaps our English sisters are taking some interest in the Superintendents' Association, if so they will note that the annual meeting fixed for February 12th and 13th, 1895, will be held in Boston.

Amongst the recent appointments announced, Miss Louisa Moss is made Superintendent of the Muhlenberg Hospital, Plainfield; and Miss Marion Williams has become Directress of Nurses at the French Hospital, San Francisco. She received her training at the Children's Hospital in that city.

### Notes from St. Helena.

BY ROSE A. BLENNERHASSETT, LADY SUPERINTENDENT OF THE CIVIL HOSPITAL.

### III.—ROUND THE WARDS WITH THE COLONIAL SURGEON.

WE went round the wards with the colonial surgeon for the first time on New Year's Day. We seemed walking into a bottomless pit of confusion and muddle. There was no orderly, for Christmas had been kept, not wisely, but too well, and complaints made to the Governor, so the orderly had been suspended for a week. He came back in a day or two, protesting that "'Twas a poor heart that didn't rejoice at Christmas," and we were very glad to have him, *faute de mieux*. He had been a smart man once, when the 48 beds of the Civil Hospital were almost always full, and a proper staff was kept up. But he was now past 60, and had had ample time to forget much of what he had learned. No one had been regularly in charge of the hospital for a considerable time, and he had therefore had things entirely his own way—a very easy going one. An Englishman would have probably resented losing his independence, and have made himself disagreeable—not so the smooth islander, with his touch of eastern suppleness. We have always found the St. Helenian pleasant mannered, amiable, unreliable, and the orderly was no exception to the rule. Although we learnt that there were rarely more than 16 patients in hospital, and it was only in exceptional cases that the 32 beds were filled, yet it was plain to see that the place would be difficult to keep clean, owing to the inconvenient arrangements.

### DRAWBACKS OF A COOK-NURSE.

For instance, the "cook-nurse," whose wards were at the top of the house, was expected to scrub them, as well as two flights of stairs and a landing, to carry slops down four flights, attend to the female patients, and do all the cooking, besides wringing out soiled sheets and blankets before sending them to the wash. It was not very surprising that her floors should be somewhat dirty. When these had been stained and beeswaxed the work was much lightened. Also the stain, composed of a strong solution of permanganate of potash, deodorised the wards.

My "cook-nurse" was obliging, willing, and good to the patients, yet, as a nurse practically in sole charge for more than a year, she had some drawbacks. She could neither read nor write, did not know the clock, or understand weights and measures! Curious to see in what manner the nursing would be carried out under these circumstances, an opportunity was taken for forming an opinion on the subject.

### A NOVEL STEAM KETTLE.

An idiotic child was admitted to hospital in a semi-unconscious state, with very bad tonsillitis, and a temperature above 104 degrees. The colonial surgeon ordered a tent, a steam kettle, inhalations, &c.

We asked if this treatment had ever been carried out before. "Oh, yes! sister-ma'am," was her answer, "I've had a-many of them, and nasty tiresome things them doctor's tents are." So we begged her to make her usual arrangements, and when we went up to the ward the child's bed was entirely concealed from view by blankets hung upon screens, a couple of blankets forming the roof of the tent. Where was the kettle? To our horror and dismay a small American kerosene oil-stove was lighted, and standing on the bed itself! A saucepan without a lid, filled with boiling water, was steaming on it!

One sudden movement of the child, who was completely out of sight from the ward, must have upset the light stove, deluged the bed with boiling water, and in all probability set fire to the sheets.

The stove and saucepan were swept away and the danger of fire spoken of, and then we asked if this arrangement had ever led to an accident? "You're right, ma'am-sister," was the reply; "the bed went on fire once, and the mother was that angry I couldn't quiet her, and all the mothers, they did say they'd never have thought a nice spoken young gentleman like the doctor, and own son to the bishop, would want to burn the children alive!"

### Private Nursing at Home and Abroad.

By a Nurse.

We venture to think that the condition of this branch of nursing is less satisfactory just now than any other. Whilst the doctors speak cordially of the advantages of having their work so ably seconded by the skilful attendant who understands their wishes, there yet remains much to be done before the ideal private nurse becomes univeral.

"By Them We Are Judged"

remarked recently, with a weary sigh, the successful head of a training school. And then she explained how, one after another, her well-taught, well-mannered ward nurses gradually deteriorated after a period of unsupervised "private work." And indeed now, whenever the word "nurse" happens to be mentioned in society, somebody is certain to volunteer a story more or less discreditable to the profession at large.

Personal Selfishness seems to be at the root of the matter, and in the notoriety attached to most modern nursing matters one type of woman is apt to convey the impression that "cases" are created for her special benefit! Therefore she amasses all she can in fees and in comfort at each private house, greatly to the detriment of unselfish nurses who follow. Mrs. Gamp made "the nurse" of her day an object of terror to the sick of her period, and when she was succeeded by the quiet, modest, neatly-uniformed figure fresh from hospital, a pioneer of better things, the public rejoiced and congratulated itself, but her successors are not all like her.

"On Her Own Account"

is the ordinary description of the nurse who "takes her own fees" and "keeps herself," and both proceedings have the cordial approbation of the thoughful. Why, then, are the results sometimes disastrous? The three years' certificate and the hospital testimonials all go to prove that a nurse has gone through the course creditably, sometimes indeed with great honour; but these things do not avail to prevent her in a few months breaking out into coloured blouses, instead of her neat uniform, and adopting a "tousled" style of hair-dressing, and a hat! Worse still, she makes herself exceedingly disagreeable to her employers if "the case" proves uninteresting or her personal comforts are not looked on as of primary importance.

A Reaction.

Doubtless this phase will be succeeded by a pleasanter one, and those excellent private nurses who make duty to the patients their first consideration will increase in numbers, but the present transition stage is certainly disagreeable. It is the private nurse alone who penetrates within the family circle, and who gives and leaves there the impression which is eventually harmful or beneficial to the whole world of nurses. Truly we may echo regretfully by "them we are judged."

Earnings of Private Nurses.

To secure to each nurse her own earnings was the object of the Nurses' Co-operation, started at 8, New Cavendish Street, and not only to the members of that association has this been done, but also to a vast number of other private nurses. Public institutions have improved the pecuniary position of their workers, and £40 per annum to a fully-qualified and experienced nurse, with uniform, board, and lodging between her cases, and kindly care in sickness, is the satisfactory outlook at several such. This, of course, compares favourably with the position of an independent worker earning two guineas or more a week, and having to board and lodge herself between her cases and also to provide herself with uniform. The old saying, "Quick come, quick go," is sadly verified in the case of the modern private nurse. Comparatively few of them make any provision for their future, such as their sisters in hospitals are encouraged to do. Luxurious personal habits and the growing love of inappropriate extravagance of dress are keeping many women's purses as ill-lined as when they were employed at £20 per annum and "everything found" for them.

Private Nurses Abroad.

Many inquiries come to us respecting the prospects for nurses abroad; these must, to a great extent, be dependent on the visitors of the locality chosen, yet a great deal is in the nurses' own hands. We are not considering the cases in which nurses go abroad with patients, their expenses and fees being then assured, for these need no advice. We strongly recommend everyone to make full inquiries as to the nursing institutions already established, and the chances of getting employment before risking the expenses of a journey and maintenance in a foreign land whilst looking out for work. It is common to hear much abuse lavished on private institutions for giving comparatively low salaries to the nurses whom they engage "for the season;" but it is well to remember that the whole expenses of getting out the nurses and keeping them is undertaken by those who cannot tell beforehand whether there will be any employment forthcoming for the people they have taken the risk of securing. A winter in the sunny South, even when spent chiefly with invalids, has many pleasant and novel experiences for the untravelled nurse who elects to try it "for a change." But we do not advise any one to go out solely in the hope of taking large fees.

### "Truth" Toy Competition.

Visitors to this wonderful exhibition last week will have owned, one and all, that the show of dolls and toys for distribution in metropolitan hospitals and workhouses for Christmas, 1893, has surpassed that of any previous year. Six huge pyramids of dolls were arranged in the centre of the Albert Hall arena, while clustered thickly round them were hundreds more, many of them life-sized, dressed in bewildering variety of costume. Enclosing the arena was an enormous bank of toys of every description, some 23,000 in all, calculated to delight and amaze all childish hearts.

One could only look with wonder on the dainty beauty of these make-believe children, over 4,000 of them, and rejoice to think of the pleasure in store for the little ones in our hospitals and workhouses, whom they will have reached ere this. A quaint figure, "In Maiden Meditation," all white satin and violets, attracted much admiration; and among others, far too numerous to mention, "A Brabant Skating Girl," dressed by Mr. C. H. Burnand, after a picture by George Boynton, won our admiration. A lovely group came from Mrs. Truefitt. The Empress Josephine, in gold and silver brocade, dressed by the Misses Hughes, was charming, and another dainty figure represented the youthful Queen of the Netherlands in national costume, contributed by Mrs. M. Peet. Again has an anonymous donor contributed 11,000 newly-minted sixpences for distribution amongst the children in the metropolitan workhouse schools, and Mr. Tom Smith sends 22,000 crackers.

The history of the *Truth* Doll and Toy Show is interesting. It was a happy thought which started it on modest lines in 1880 in two small rooms in *Truth* offices in Queen Street, and year by year has the success attained grown greater and greater. One place of exhibition after another has been outgrown till the Albert Hall has for the last three years been kindly placed at the disposal of the editor of *Truth*, by the Executive Council. From 1,000 toys in 1880 the grand total has reached nearly 28,000 this year. Many visitors thronged the Hall last week, and we hope many were induced to drop some contribution into the boxes and bags which waited for them. Unfortunately there was a deficit of nearly £300 last year, and we hope the special efforts being made to reduce this amount may be entirely successful. It would be a calamity indeed should anything occur to render possible the discontinuance of a scheme which brings so much pleasure into the lives of children to whom poverty and suffering are familiar, and enjoyment, as understood by their more fortunate brothers and sisters a thing almost unknown.

### Everybody's Opinion.

[Correspondence on all subjects is invited, but we cannot in any way be responsible for the opinions expressed by our correspondents. No communications can be entertained if the name and address of the correspondent is not given, or unless one side of the paper only be written on.]

### SOUTH AFRICA AS A HEALTH RESORT.

MISS ROSE A. BLENNERHASSETT writes from St. Helena: I was much interested in "P. D. M.'s" letter on this subject, which appeared in THE HOSPITAL of October 20th. Your correspondent accurately describes the difficulties which beset the path of the health seeker in South Africa, and I am in receipt of several letters on this subject, written by doctors at present practising in South Africa. All declare that the benefit obtainable from the pure dry hot African air cannot be over estimated; and all deplore the absence of suitable arrangements for the comfort of invalids. Writing from a health resort in South Africa a well-known doctor says: "The air here is perfect—dry and bracing. This place lies some 3,800 (4,000) feet above the sea level. Last year I attended 16 patients who were obliged to stay at the hotel. Some eight or twelve of them left greatly disappointed, as they received no comfort." The hotel diet, he adds, is all very well for healthy people, but as most cases of lung diseases are associated with a certain amount of dyspepsia, it is easy to understand that careful feeding is most essential for them, and that without it they cannot expect to gain weight. The doctor writes from a place highly recommended by Dr. Symes Thompson. Sister Lucy Sleeman and I are leaving St. Helena, and propose to open a home for phthisical patients in Africa. We shall offer them careful nursing and home comforts at prices that will not be prohibitive to those who are not rich people. We intend to keep our own cows and poultry, grow our own vegetables, and therefore hope to be able to supply invalids with an abundance of the sorts of food they need without extra charge. We hope to carry out our plan in January-February, 1895. Beginning on a small scale as an experiment, we may, of course, end in utter failure, but neither of us believes in failure. I will say no more until we are established in Africa, when I will send full particulars in the hope that the Editor will give our small scheme the "backing up" of THE HOSPITAL by making it known to the medical and nursing world.

### NURSING IN THE RIVIERA.

A TRAINED NURSE AND SUPERINTENDENT, writes: Seeing often in THE HOSPITAL, queries as to nursing on the Riviera, perhaps a few statements from one who has had long experience there may prevent disappointment to those wishing to come out to nurse "on their own account."—Every town of any size has now a Nurses' Institute, to which the English doctors as a rule promise their support. Therefore, as long as the institute can supply them, the outside nurse has no chance whatever. In many places there are also nurses who have worked for years under one doctor, and in so doing have worked up a connection for themselves. A nurse "run down" in an English hospital may be told "Why not try a winter on the Riviera? There is plenty of work, directly you are known you will have more than you can do." The nurse therefore starts, and, arriving at her destination, interviews the doctors, only to be told that they are pledged either to the institute of the town, or to nurses they know personally, and have employed for years. If, as in the last three seasons, influenza should be rife, for a week or two a stranger may get a case. But awaiting this possibility, five out of the six months of the season pass in "hope deferred." Doctors and matrons of institutes are besieged with applications not only from nurses in England wanting to come out, but from those who have come without counting the cost, and find themselves richer in experience but, alas! poorer in pocket for their venture. Having made their own arrangements for the winter, both doctors and matrons are unable to help, the supply of nurses far exceeding the demand. With regard to ways and means—unless a nurse can afford to spend at the least £2 a week for living, she had better stay in England, taking a holiday there, and saving the £12 for the journey out and home. To those who cannot afford to "play a waiting game," I would say apply to the matron of some institute, at the end of one season, to be engaged the next—stating clearly all particulars, age, training, &c. Without these details many letters remain unanswered. It is impossible that endless replies should be sent at 2½d. postage, and matrons often receive three or four applications a week throughout the season.

### A CORRECTION OF "A CORRECTION."

MR. T. Vincent Jackson, F.R.C.S., writes: "In your issue of December 15th you were good enough to insert a paragraph announcing the opening of a Home for the District Nurses in Wolverhampton, which had been taken and furnished in a very complete way by the Queen Victoria Nursing Institute. Unfortunately, your announcement made it appear that the new Home was "the Wolverhampton branch of the Queen's Jubilee Institute"; this, of course, was incorrect, and of which Miss Peter hastened to inform you. The error, which was not ours, was unknown to us, and had Miss Peter simply corrected it, the matter would have ended. Miss Peter, however, in her "correction," goes on to say that the "new Home opened November 22nd, and called the Queen Victoria Nursing Institute, is for private nurses only. The two District Nurses for Wolverhampton live in lodgings in different parts of the town." As these statements of Miss Peters' are absolutely incorrect, I must ask her, through you, to withdraw them.

### KIMBERLEY HOSPITAL.

DR. DRAPER BISHOP writes: Since the last notice relating to this hospital appeared in your paper, I learn that the Hospital Board . . . have declined to allow Sister Henrietta to call her private staff of nurses "The Kimberley Hospital Private Staff," or to refer to them as being in any connected with the hospital.

### Nursing in Holland.

THE Society for the Christian Care of the Insane in the Netherlands has made an extensive purchase of land in the neighbourhood of Zuidlaren, in the province of Drenthe, for the erection of an asylum. The ground is well covered by fir plantations, and in the course of last winter roads and paths were laid out, and it is hoped that the buildings will be completed and the institution ready in the course of 1895. The same society has also obtained permission to build an asylum for the insane of the higher classes at Ermeloo.

On October 30th last the ceremony of distribution of the "White Cross" diplomas took place at the Wilhelmina Hospital; formerly they were despatched by post to the successful candidates, but this year it was determined to make an alteration in the method of procedure. The hall of the hospital was decorated for the occasion, and the president, Dr. Barnouw, in the presence of the committee and numerous spectators, handed the diplomas and gave a short address to the recipients.

The Utrecht Society of Deaconesses has just celebrated its fiftieth year of existence, and the occasion was celebrated by a very successful bazaar, the deaconesses and other ladies acting as stall-holders. A crowded meeting was held, at which delegates from different parts of the country were present, and congratulatory letters not only from Holland, but from France, Germany, and Switzerland were read. Much enthusiasm was aroused by the announcement of a gift of five hundred francs to the institution from her Majesty the Queen of Holland.

The society for providing the hospitals of Amsterdam with flowers, fruit, and books, has just issued its twelfth report. Especial thanks are given not only to those who have helped the society with gifts, but also to the hospital sisters who have undertaken the distribution and care of the flowers.

### Christmas Books.

"Lerne zu leiden ohne zu beklagen"—bear without grumbling—is the motto all try to put in practice, if only for a few hours on Christmas Day. And it is pleasant to note the changes rung upon this motto in the books which cumber the study table at this season. Readers of the "Memoir of Sir Morell Mackenzie," written by Mr. Haweis, will remember the words as favourite ones of the great Emperor Frederick, and as a leading note in the life of the physician whose name is inseparably linked with his. The new edition,* lately issued, of this memoir, recalls under what constant pressure of chronic suffering Sir Morell Mackenzie worked through his arduous days, and the high value he set on resolute disregard of pain in friends and patients. It is chiefly from this point of view, indeed, as the unfolding of a personality possessed of singular attraction to the inner circle of friends, that the memoir can claim attention. The time has not yet come for a full discussion of the great controversies upon which Mr. Haweis touches with an uncertain hand, but friends and foes alike, and never was a man who could boast a more generous share of both, can profit from this picture of lifelong struggle and mastery over sleeplessness and pain which in most people would constitute an excuse for chronic invalidism.

Father Thomas Burke, O.P., was a very different man in almost every respect from the man of science, but his memoir,† by a Dominican friar, suggests a similar train of thought, and reveals a similar conquest of indomitable will over ceaseless bodily pain. And it is very striking that the great preacher, whose fame was in every mouth, and whose personal sanctity was a rebuke to his associates, should have found in his Irish gift of humour his happiest weapon against self-pity and surrender. He fully bore out his own *bon mot*—"There is no law that good people should be stupid; they might be Sankeymonious without being Moody." Many instances are recorded of his wonderful command over pain. "Preaching was his vocation and his greatest delight. Yet it was when in the pulpit that he felt the acutest pain. The beads of perspiration stood out upon his forehead and rolled down his face, evidence of the agony he was undergoing. . . . After superb displays of oratory, he dragged himself back to his bed of ceaseless pain, there to regain strength for the next effort." And again, "Even when prostrate with pain he would drag himself to the recreation-room, and by a considerable effort would throw off his sick self and resume the buoyant spirit of his younger and more healthful days." The self-discipline involved in preaching under such circumstances is, perhaps, even less remarkable than the wit which could turn his pains into puns and set a sympathising circle of bystanders laughing against their will in the midst of one of his worst paroxysms. Alluding to the saints of old, he wrote, "Did they ever flinch, those magnificent servants of God?" and we may well echo the words when we look into the narratives of noble lives and brave deeds collected by Mr. Cross under the title of "Beneath the Banner."‡ A good little book for those whose time for reading is limited, or for those who cannot bear the strain of a long narrative, these little biographies may fill up many a weary or aimless ten minutes and leave plenty of food for meditation afterwards. They are simply written, and all contain in their short compass some fresh illustration of joy in self-surrender and contempt of pain.

* "Sir Morell Mackenzie." By the Rev. H. R. Haweis. (W. H. Allen and Co.)

† "Inner Life of Father Thomas Burke, O.P." By a Dominican Friar. (Burns and Oates, Limited.)

‡ "Beneath the Banner." By E. J. Cross. (Cassell and Co., Limited.) Price 1s.

### For Reading to the Sick

CHRISTMAS.

**Motto.**

Let not the hearts, whose sorrow cannot call
This Christmas merry, slight the festival;
* * * * *
For the *weepers* was the Saviour born.
—*H. Coleridge.*

**Verses.**

Thou cam'st from heaven to earth that we
Might go from earth to heaven with Thee;
And though Thou found'st no welcome here,
Thou did'st provide us mansions there.
—*H. Vaughan.*

Wrapped in His swaddling bands,
And in His manger laid,
The Hope and Glory of all lands
Is come to the world's aid. —*J. Keble.*

One Name alone in all this death-struck earth,
One Name alone came down from highest heaven,
Whence healing and salvation we receive,
To sinful man is given.
O Name of Jesus!—of that lowly Babe
That on the sunny slopes of Nazareth strayed,
Or, calm and silent on the cottage floor,
With wild flowers played:
Name of the wondrous Child, . . . .
Name of the Prophet, Healer, Master, Friend,
Death's mighty Vanquisher, and Sorrow's Cure,
The Fountain of new innocence for man,
That ever shall endure.
Name that the ransomed souls for ever wear,
Gemmed with pure lustre on each perfect brow,
Be Thou the radiance of our earthly lives;
Transform us even now.
—*Caroline Noel.*

**Reading.**

"And when the star appeared they rejoiced with exceeding great joy." The pilgrimage of faith, like the journey of the wise men, is not always plain and easy. The full light of Heaven does not come until we are ushered into the presence of the King. . . . When faith first begins to live and use its powers within the soul, it is as the rising of a new and unknown star. How the knowledge of God's love, in sending a Saviour into the world, seemed to gleam and burn in the deep blue heavens, as if to say, that it had but one message for one soul, and now the sad heart was all at once filled with a joy that it had not before conceived. Since then, perchance, the pilgrimage has been long and weary, the path of duty has not always been illumined from above, trials and dangers have beset the way—but lo! after life has advanced the star appears again, and it is to be hoped with still greater brilliancy, not now afar off, speak of light glistening at the distance, but hovering near us, and leading us on, until Christ is revealed to us as the supreme object of our love and worship, leading us on until faith will be no longer needed, and starlight will give place to the glorious beauty of the Sun of Righteousness. —*H. M. Neville.*

He has come to wipe away our tears, bear our sorrows, to cheer our sad hearts, and fill them with the joy which at Christmastide comes to all Christ-like souls. —*Anon.*

### Dress and Uniforms.

By a Matron and Superintendent of Nurses.

### Specialities in Linen.

It is needless to say that everything kept by Messrs. Walpole and Sons is of the best quality, both as regards texture and finish. Irish linen has long maintained, and justly so, a reputation for excellence; and to bring it within the grasp of the British public, Messrs. Walpole import the genuine article direct from their headquarters in Ireland, and offer it at reasonable prices to the discriminating purchaser at their emporium at 89, New Bond Street. A pure white linen of admirable quality 40 inches wide would prove invaluable for aprons, as well as pillow cases, and the price is low enough to tempt all genuine lovers of good material. This quality can be had in all widths at a small corresponding increase of cost, which greatly enhances its usefulness. Their Diaper kitchen rubbers will be found quite a luxury, they are so soft, and yet have the appearance of being both strong and durable. Fine glass cloth can be purchased at a price as moderate as that paid for the numberless inferior makes which at present flood the market. The same remark applies to the damask table cloths, dinner napkins, and linen sheetings, all of which are warranted pure linen made from flax, and are deserving of the highest recommendation.

### Nursing Uniforms at Messrs. Derry and Toms.

Those among our readers who are dwellers in Kensington will be glad to hear that Messrs. Derry and Toms have opened a department for nursing uniforms at their spacious premises in High Street. The name of this firm is sufficient to guarantee excellence both of material and workmanship in all they undertake, and it will be found that the uniform falls in no way short of their average. Especially worthy of notice are the plain circular cloaks, which can be had in all colours, black and blue, however, being kept in stock. The shape is good, and the material can be supplied in either fine serge or thick cloth, according as to whether it is required for winter or summer wear. A little open "Princess" bonnet in a soft shade of grey, trimmed with velvet, and with a gossamer fall of the same colour, has a very charming appearance. One similar in navy blue is also pretty, the brim is turned up with velvet, and a large bow of ribbon is placed in front. A neat little border of fine net, adds a becomming finish to both these bonnets. Dress material is offered in a variety of shades and patterns, and estimates of made up costumes, &c., can always be obtained on application. Messrs. Derry and Toms pay special attention to all country orders, and will be pleased to forward a catalogue post free to any address.

Nurse's Bonnet.

### A Boon to Nurses.

Foremost among those benefactors to nurses who suffer with their feet, must be mentioned Messrs. Elliott and Sons, of 106, Westbourne Grove, who have devised a boot which is admitted by all who have given it a trial to be one of the most effectual counterfoils to flat foot that has hitherto been designed. A flexeura spring is introduced into the sole just under the instep, which prevents it falling, and at the same time supports it so firmly, that the sufferer is enabled to pursue her duties without any inconvenience whatsoever. The boot is buttoned, and so ingeniously contrived that, in addition to the advantages already enumerated, it has the further one of causing the foot to appear at least two degrees smaller than its natural size. This happy result is attained by the arched sole, which is thus made to serve a twofold purpose. Then there is a walking shoe constructed on a similar principle, which will prove a real boon to those who have neither the time nor the inclination to go through the buttoning process, unavoidable where boots are concerned. Messrs. Elliott also make a very nice kind of ward shoe which buttons with a strap over the instep and is capped with india rubber to deaden sound, the heels are square and of medium height, and the sole broad and well shaped. All these goods are hand sewn, and being cut out of well-seasoned glacé kid, are not only beautifully soft and comfortable to the foot of the wearer, but exceedingly moderate as regards price.

### Novelties at the Boston Rubber Company.

Galoshes are never, under any circumstances, the most becoming adjunct to the foot, but the uncertainty of our climate makes them a necessary evil. No one, however, who has to be out in all weathers will venture to dispute either their usefulness or their convenience. The Boston Rubber Company, by the excellence of their shapes and the lightness of their material, will do much to reconcile the public to the use of a rubber. Unlike the old-fashioned galoshe, space is provided for the heel of the shoe, which, of course, is very much more becoming to the foot than when made perfectly flat. A delightful little boot for a child is fashioned with a strong rubber sole and angola cloth uppers, which will ensure the foot within being kept dry, even in spite of a youthful predilection for puddles on a wet day. A tennis shoe in white canvas will be found equally useful, being provided with a light though strong indiarubber sole. Most comfortable to invalids with a gouty tendency are the stout cloth boots laced down the front supplied by this firm. The sole is very broad and flat, allowing for the free expanse of the foot, while the cloth tops are charmingly soft, and constructed in such a manner as to give no undue pressure on any of the prominences. The Boston Rubber Company are to be congratulated on their successful effort to meet that for which there must be always more or less of a demand.

### Presentations.

The managers of the Western Infirmary, Glasgow, have presented to Dr. Mackintosh on the occasion of his marriage a silver salver and cake basket, and to Mrs. Mackintosh they have given a diamond ring. The visiting medical staff have given a chased silver bowl, and the resident medical officers, the nursing and domestic staff, with the male employés of the infirmary, have all given handsome testimonials. Dr. Mackintosh was a graduate of Glasgow University, resident surgeon at the Eye Infirmary, resident medical officer at Belvidere Fever Hospital, superintendent of the Victoria Infirmary. He is now superintendent of the Western Infirmary, where he is held in deservedly high esteem.

### A Special Hospital.

The committee of the Tunbridge Wells Eye and Ear Hospital appeal for aid to raise a special fund of £200 to meet the increasing number of patients and also to pay for some needful structural alterations. 649 out-patients and 73 in-patients were treated last year.

## Figures and Tables

**Figure f1:**
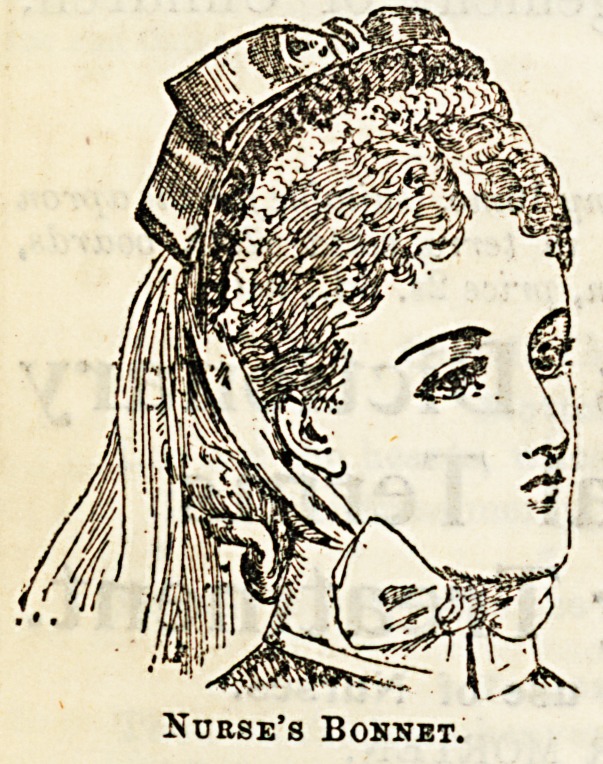


**Figure f2:**
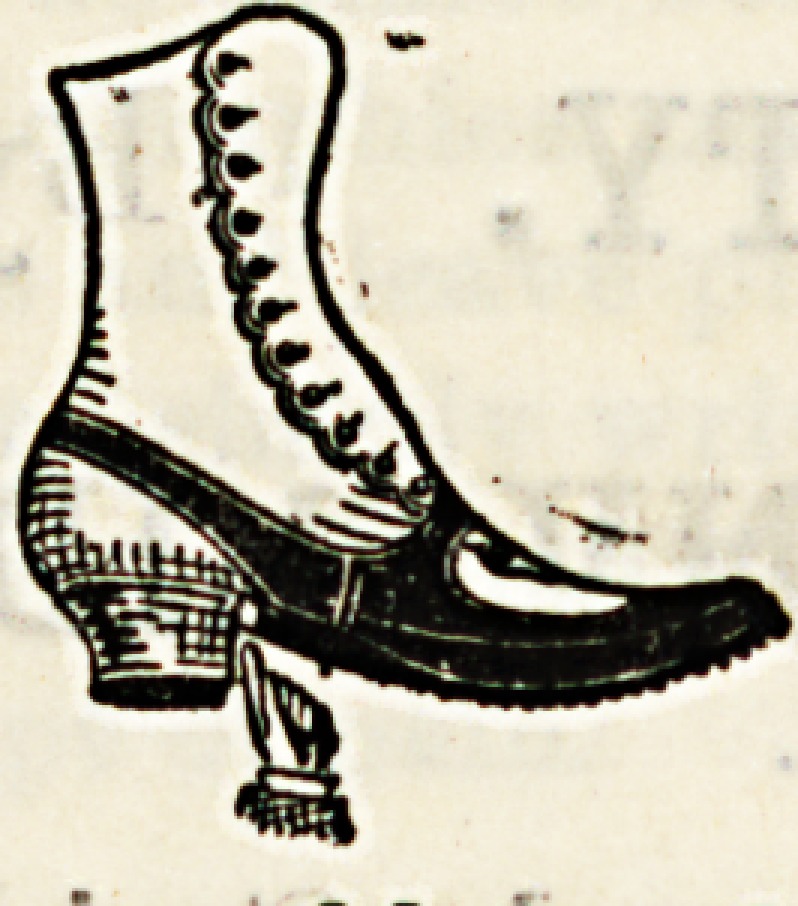


**Figure f3:**
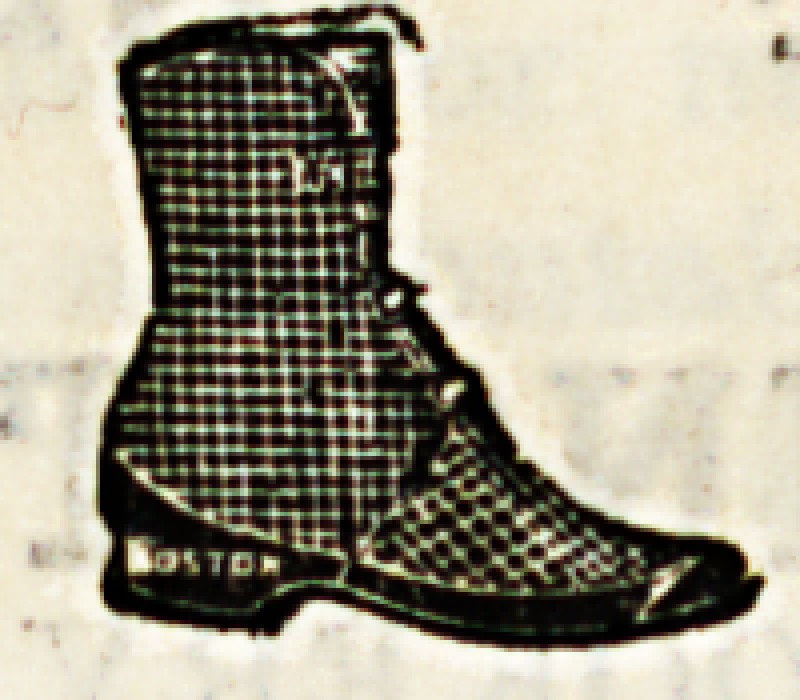


**Figure f4:**